# Public attitudes towards screening for kidney cancer: an online survey

**DOI:** 10.1186/s12894-020-00724-0

**Published:** 2020-10-28

**Authors:** Laragh L. W. Harvey-Kelly, Hannah Harrison, Sabrina H. Rossi, Simon J. Griffin, Grant D. Stewart, Juliet A. Usher-Smith

**Affiliations:** 1 University of Cambridge School of Clinical Medicine, Addenbrooke’s Hospital, Hills Road, Cambridge, CB2 0SP UK; 2 The Primary Care Unit, Department of Public Health and Primary Care, University of Cambridge, Cambridge, CB2 0SR UK; 3 Department of Oncology, University of Cambridge, Addenbrooke’s Hospital, Cambridge Biomedical Campus, Hills Road, Cambridge, CB2 0QQ UK; 4 Department of Surgery, University of Cambridge, Addenbrooke’s Hospital, Cambridge Biomedical Campus, Hills Road, Cambridge, CB2 0QQ UK

**Keywords:** Attitudes, Kidney cancer, Cancer, Screening, Survey

## Abstract

**Background:**

Kidney cancer is often asymptomatic, leading to proposals for a screening programme. The views of the public towards introducing a new screening programme for kidney cancer are unknown. The aim of this study was to explore attitudes towards kidney cancer screening and factors influencing intention to attend a future screening programme.

**Methods:**

We conducted an online population-based survey of 1021 adults aged 45–77 years. The main outcome measure was intention to attend four possible screening tests (urine, blood, ultrasound scan, low-dose CT) as well as extended low-dose CT scans within lung cancer screening programmes. We used multivariable regression to examine the association between intention and each screening test.

**Results:**

Most participants stated that they would be ‘very likely’ or ‘likely’ to undergo each of the screening tests [urine test: *n* = 961 (94.1%); blood test: *n* = 922 (90.3%); ultrasound: *n* = 914 (89.5%); low-dose CT: *n* = 804 (78.8%); lung CT: *n* = 962 (95.2%)]. Greater intention to attend was associated with higher general cancer worry and less perceived burden/inconvenience about the screening tests. Less worry about the screening test was also associated with higher intention to attend, but only in those with low general cancer worry (cancer worry scale ≤ 5). Compared with intention to take up screening with a urine test, participants were half as likely to report that they intended to undergo blood [OR 0.56 (0.43–0.73)] or ultrasound [OR 0.50 (0.38–0.67)] testing, and half as likely again to report that they intended to take part in a screening programme featuring a low dose CT scan for kidney cancer screening alone [OR 0.19 (0.14–0.27)].

**Conclusion:**

Participants in this study expressed high levels of intention to accept an invitation to screening for kidney cancer, both within a kidney cancer specific screening programme and in conjunction with lung cancer screening. The choice of screening test is likely to influence uptake. Together these findings support on-going research into kidney cancer screening tests and the potential for combining kidney cancer screening with existing or new screening programmes.

## Background

Internationally, there is great interest in evaluating the potential for a screening programme for kidney cancer [1–3]. Half of all patients with kidney cancer have asymptomatic disease and up to a quarter of individuals have evidence of metastases at diagnosis [4]. Survival is strongly related to stage at diagnosis [4]. A screening programme, potentially in a selected higher risk population, may down-stage the disease, reduce the prevalence of metastatic tumours at diagnosis, improve survival and decrease the expenditure related to systemic therapies [5].

The two currently available potential screening modalities are ultrasound and CT scan. Our group reported a detailed cost-effectiveness analysis of ultrasound scan-based screening. This suggested that screening may have acceptable cost-effectiveness in men aged 50–60y (£18–22k/QALY) and in women aged 60 if the prevalence of kidney cancer is at least 0.2% [6]. When combined with other CT scan-based screening programmes, such as current lung cancer screening pilots [7], additional screening for kidney cancer by CT scan has the potential to provide health and economic benefits. Research into new urinary (i.e. perilipin 1 and aquaporin 2 [8]) and blood based markers (i.e. KIM-1 [9]) is also on-going and these may provide additional options for screening in the future.

Whichever screening modality is used, the benefits will only be realised if sufficient numbers of the target population undergo screening. This requires consideration of the views of the population on the acceptability and potential engagement with future programmes [10]. To our knowledge, no studies have addressed these issues for kidney cancer. We aimed to explore the attitudes of the public towards potential future screening programmes for kidney cancer with different tests and to understand the individual and system-level factors influencing intention to take up screening.

## Methods

### Study design

We conducted an online population-based survey. The survey was conducted and is reported in line with the checklist for Reporting Results of Internet E-Surveys (CHERRIES) [11].

### Participants and recruitment

We aimed to recruit 1000 participants (50% female) through Prolific (www.prolific.ac), an online platform in which individuals volunteer to take part in studies and are compensated for their time at an hourly rate (£6/hour). Participants were eligible if they were between 45–79 years of age and, in line with accepted practice [12], had a Prolific approval rating ≥ 95%. The approval rating reflects the percentage of studies that participants have completed that have been approved by researchers. We chose the age range 45–79 years because it is unlikely that a future kidney cancer screening programme would screen individuals younger than 45 and we wanted to be able to capture any differences in attitudes in older groups. We did not prevent participants with a history of kidney cancer from completing the survey but their data were subsequently excluded from the analysis.

When the survey was launched, a random subset of eligible participants were invited to complete the survey via email through the Prolific platform (Additional file 1). The survey then remained open to new participants until recruitment was complete. After starting the survey, all participants had the option to ‘return’ their submission at any point. This allowed them to stop part way through the study without affecting their Prolific rating. Data from participants who returned their submission were deleted and not analysed. Cookies were not used to identify each participant computer. Duplicate entries were prevented within the Prolific platform through use of unique participant identification numbers.

### Survey

The survey was developed by the study team and piloted with two patient and public representatives to check that the questions were comprehensible and being interpreted correctly. Validated questions were used wherever possible. A complete version of the survey is provided in Additional file 2. Briefly, participants first provided socio-demographic details including age, lifestyle, highest level of education, employment and socioeconomic status (assessed via the occupation of the household’s chief income earner [13, 14]). They were then asked questions about their medical screening history, perception of cancer risk, cancer worry [15], knowledge of kidney cancer symptoms and risk factors for kidney cancer [16]. Participants were then given brief summary information about kidney cancer and the estimated benefits and harms of four potential screening methods using data from the literature [5]. A series of questions followed about beliefs and attitudes concerning kidney cancer and screening [17] and whether, based on the information provided, participants would take part in screening. Finally, participants were asked about how much influence various other factors, identified from the literature [18], would have on their decision to undergo screening. Written online consent was obtained from each participant before they began the survey.

### Data collection

The questions and scenarios were embedded within an online questionnaire and two studies (one for men and one for women) run in parallel on the Prolific website to enable us to achieve a sample with equal numbers of men and women. All questions except those inviting free text responses were mandatory for all participants. The questions were distributed over 22 pages with between one and seven questions per page to enable completion on tablets or smartphones with small screens. It was not possible for participants to go back through the pages and change their answers. The technical functionality of the electronic questionnaire was tested by the study team. We included an instructional manipulation check to identify inattentive participants and increase the validity and reliability of the responses [19]. Participants who failed to answer this question correctly were excluded from the study.

### Analysis

We used descriptive statistics to characterise the participants and their overall attitudes towards, and intention to take-up, kidney cancer screening. Socioeconomic status (SES) was classified as higher (ABC1) and lower (C2DE). For awareness of kidney cancer symptoms and risk factors participants were categorised into two groups (high/low) based on whether their overall score was above or below the median (see Additional file 2 for details).

We used a linear regression model to analyse differences between the burden/ inconvenience and worry associated with each screening test. The null hypothesis of no difference between the four groups was tested using an F-test, followed by the estimation of four pairwise contrasts between the screening tests: (1) urine vs blood; (2) urine vs ultrasound; (3) urine versus CT; and (4) ultrasound vs CT. Confidence intervals (98.75%, based on a Bonferroni corrected significance threshold of 1.25%) are presented for these analyses to acknowledge that four pairwise comparisons were performed.

Logistic regression was then used to examine the influence of the different screening tests and participant characteristics on intention to take up screening. For these analyses, cancer beliefs about treatment was treated as a continuous variable from 1–5, 1 reflecting the view that most cancer treatment is worse than the cancer itself. Cancer beliefs about outcomes was similarly treated as a continuous variable, computed by combining responses from the relevant questions 1, 4 and 6 using published coefficients [17] (see Additional file 2 for details).

To enable us to compare the intention to take up each of the five screening test modalities, we included the screening test as an independent variable as well as adjusting for participant characteristics (age, sex, education level, ethnicity, self-reported general health, country of residence, income group, body mass index (BMI), smoking status, prior history of cancer, family history of kidney cancer), cancer worry, perceived risk of cancer, beliefs about cancer outcomes and treatments, the mean burden/inconvenience and the mean worry associated with all of the tests combined for each participant. The analysis was adjusted for the clustering of responses by participant using the—cluster—command in Stata. To enable comparison of participant characteristics associated with intention to take up each of the screening tests separately, we repeated the analysis for each screening test with the burden/inconvenience and worry associated with each specific test. We checked for collinearity between the covariates in the regression models by calculating the variance inflation factors (vif) for each covariate using the command –collin- in Stata. All variables had vif values less than 4.20, suggesting no evidence of substantial collinearity. We also separately assessed for interactions between cancer worry, perceived risk of cancer and beliefs about cancer outcomes, and burden/inconvenience of the screening tests and worry about the screening tests by adding interaction terms into the regression. The hypothesis being tested was that worry about the screening tests and the perceived burden/inconvenience of screening may have less of an impact on intention to take up screening among those individuals with higher cancer worry, higher perceived risk of cancer or stronger negative beliefs about the outcomes of cancer. Interactions were considered significant if p<0.05. Where the interaction was significant we additionally stratified by high (> 5) and low (≤ 5) cancer worry and estimated the association between worry about screening tests and intention to take up screening separately in these two groups. All results from these analyses are presented as odds ratios (OR) with 95% confidence intervals.

All analyses were performed using Stata Version 14.

### Patient and public involvement

Two members of the public contributed, through face-to-face discussions and email, to the development of the survey and participant information sheet. They also provided comments on the final manuscript prior to submission.

## Results

At the time of recruitment, 7767 participants were registered with Prolific and met the eligibility criteria for the study. Based on demographic data on the Prolific website, approximately 80% of these were White/Caucasian, 34% male, 53% lived in the UK, 33% lived in the US, 15% were current smokers and 43% had a university level education. 51% considered themselves to be in the top five deciles of socioeconomic status. The survey was live for eight hours. During this time 1190 participants clicked through to view the participant information leaflet and consent form. 1077 (90.5%) started the survey. 29 failed the instructional manipulation check and so were excluded. A further 22 returned their submission. 1025 (86%) completed the survey. Four of those 1025 participants reported a history of kidney cancer and so were excluded from analysis. The characteristics of the remaining 1021 participants are shown in Table 1. Participants were aged from 45–77 years, 513 (50.2%) were female. Reflecting the eligible participant pool, the majority were from the UK (668, 65.4%) or USA (183, 17.9%), 46% had a university level education and 16.1% were current smokers. Men were over-represented amongst the study participants due to our sex-specific recruitment strategy. The proportion of participants of White ethnicity (95.3%) and those of higher SES (76.2%) were also higher among the study participants than the eligible population. A previous diagnosis of any cancer was reported by 63 (6.2%) and a family history of kidney cancer by 29 (2.8%). The majority (835, 81.8%) knew nothing about kidney cancer or had only heard of the condition before participating in the survey.

**Table 1:** Participant characteristics

Participant characteristic	n (%) or mean (± s.d.)
Age	
45–54	590 (57.7)
55–64	328 (32.1)
> 65	103 (10.1)
Mean (± s.d.)	54.3 (± 7.1)
Sex (n, % female)	513 (50.2)
Country	
UK	668 (65.4)
US	183 (17.9)
Europe	77 (7.5)
University level education	471 (46.1)
Ethnicity (n, % White)	973 (95.3)
General health measure	
Excellent, very good, good	811 (79.4)
Fair, poor	210 (20.6)
Smoking status	
Non-smoker	512 (50.1)
Ex-smoker	345 (33.8)
Current smoker	164 (16.1)
BMI (kg/m^2^) (mean ± s.d)	27.4 (± 5.7)
Previous diagnosis of cancer	63 (6.2)
Family history of kidney cancer	29 (2.8)
Social group*	
ABC1	778 (76.2)
C2DE	188 (18.4)
Kidney cancer knowledge	
I am very familiar with it	6 (0.6)
I have only heard of the term before	497 (48.7)
I know a little about the disease	180 (17.6)
Nothing at all	338 (33.1)
Cancer worry (mean ± s.d)**	5.6 (± 2.3)
Cancer risk perception (mean ± s.d)***	34.1 (± 23.2)
Kidney cancer symptom awareness****	
High symptom awareness (≥ 5)	547 (53.6)
Low symptom awareness (< 5)	474 (46.4)
Kidney cancer risk factor awareness****	
High risk factor awareness (≥ 43)	585 (57.3)
Low risk factor awareness (< 43)	436 (42.7)

*ABC1 = higher SES, C2DE = lower SES

**On a scale of 3–15 where 3 = no worry, 15 = maximum worry

***On a scale of 1–100 from unlikely to likely

****Where high/low awareness was defined as above/below the median score. The breakdown for each risk factor/symptom can be found in Additional file 3: Supplementary Table 1, and the scoring system in Additional file 2

### Cancer beliefs and attitudes towards kidney cancer screening

Table 2 shows the participants’ general cancer beliefs and their attitudes towards kidney cancer screening. The majority of participants were positive about cancer outcomes, only 6.7% expressed cancer fatalism*.* Attitudes towards screening were also positive, the majority (91.8%) agreed with the statement that screening could reduce the chance of dying from kidney cancer.

**Table 2:** General cancer beliefs and attitudes towards kidney cancer screening

	Strongly agree /agree (n, %)	Neither agree nor disagree (n, %)	Disagree or Strongly disagree (n, %)
*General cancer beliefs*			
“These days, many people with cancer can expect to continue with normal activities and responsibilities”*	704 (69.0)	206 (20.2)	110 (10.9)
“Cancer can often be cured”*	740 (72.5)	199 (19.5)	82 (8.1)
“Some people think a diagnosis of cancer is a death sentence. To what extent do you agree or disagree that cancer is a death sentence?”*	138 (13.5)	310 (30.4)	573 (56.1)
Beliefs about cancer treatment: “Most cancer treatment is worse than the cancer itself”	406 (40.1)	343 (33.6)	272 (26.6)
Cancer fatalism: “I would NOT want to know if I have cancer”	68 (6.7)	109 (10.7)	844 (82.7)
*Attitudes towards kidney cancer screening*			
“I would be so worried about what might be found at kidney cancer screening that I would prefer not to have it.”	72 (7.1)	83 (8.1)	866 (84.8)
“Kidney cancer screening is only necessary if I have symptom”	80 (7.8)	149 (14.6)	792 (77.6)
“I don’t think there is any point going for kidney cancer screening because it won’t affect the outcome”	13 (1.3)	71 (7)	937 (91.8)
“Kidney cancer screening could reduce my chance of dying from kidney cancer.”	937 (91.8)	45 (4.4)	39 (3.8)

*Questions that were combined using published coefficients to form “Beliefs about cancer outcomes”

### Intention to attend screening

Intention to take-up screening was high overall (Table 3), with over 90% reporting that they would accept the invitation for screening by urine testing, blood testing or low-dose CT combined with lung screening. Intention was lower for ultrasound (89.5%) and low-dose CT of kidneys alone (78.8%).

**Table 3:** Intention to take up screening and the burden/inconvenience and worry associated with each screening test

Screening test	Mean Burden/ inconvenience (mean ± sd)*	Worry (mean ± sd)*	Intention to take up screening	
			Unlikely n (%)	Likely n (%)
Urine test	1.2 (± 0.5)	1.2 (± 0.6)	60 (5.9)	961 (94.1)
Blood test	1.6 (± 0.7)	1.4 (± 0.7)	99 (9.7)	922 (90.3)
Ultrasound scan	1.6 (± 0.8)	1.3 (± 0.7)	107 (10.5)	914 (89.5)
Low dose CT scan	1.9 (± 0.9)	1.7 (± 0.9)	217 (21.3)	804 (78.8)
Low dose CT scan combined with lung screening**	–	–	49 (4.8)	962 (94.2)

*1=none and 5=very great; **10 participants did not answer this question.

**Fig. 1:**
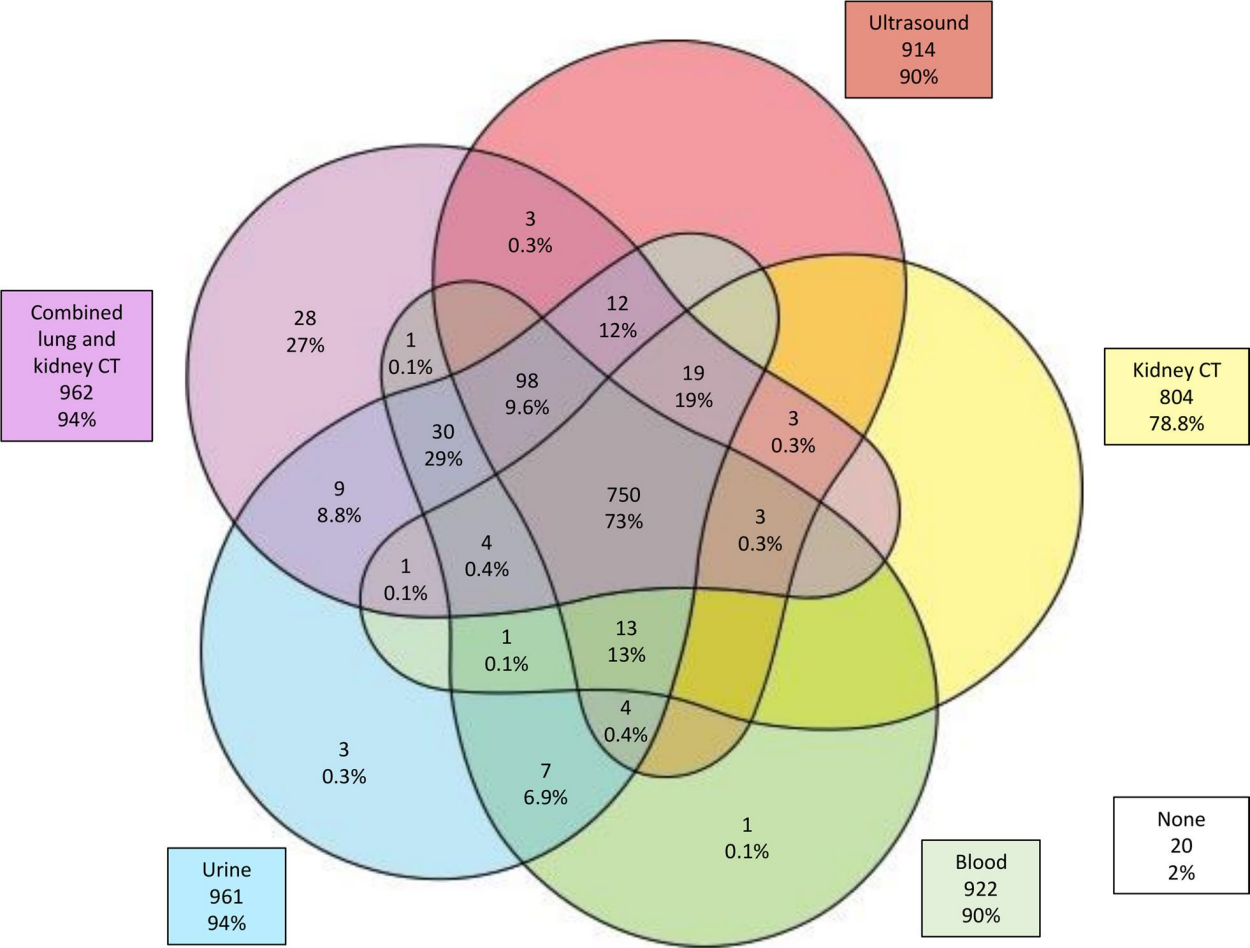
Participants expressing intention to take up screening across each of the five screening modalities. Blank areas indicate 0 participants. *n*=1021

Fig. 1 shows the pattern of responses at an individual level across the five modalities. A majority (771, 76%) reported that they would attend screening by all five modalities, 20 (2%) said they would not attend screening by any method and 28 (27%) reported they would only attend screening by the combined lung and kidney CT scan and not by the other methods. 217 (21%) participants would not attend screening by CT scan of the kidneys alone, but 181 of these 217 (83%) participants would attend kidney cancer screening by CT scan if lung cancer screening was also included.

The mean burden/inconvenience and the mean worry for each of the four screening tests are given in Table 3. Both were measured on a scale from 1–5. There were significant differences between the screening tests in both burden/inconvenience and worry, with significantly higher values for ultrasound, blood test and low-dose CT than for urine test [mean difference 0.38 (0.32–0.44) and 0.16 (0.12–0.19) for burden/inconvenience and worry respectively for ultrasound, 0.33 (0.28–0.37) and 0.11 (0.07–0.15) for blood test and 0.69 (0.62–0.75) and 0.47 (0.41–0.53) for low-dose CT]. Additionally, both burden/inconvenience and worry were significantly higher for low-dose CT than ultrasound (mean difference 0.31 (0.26–0.35) and 0.36 (0.31–0.41)).

**Fig. 2:**
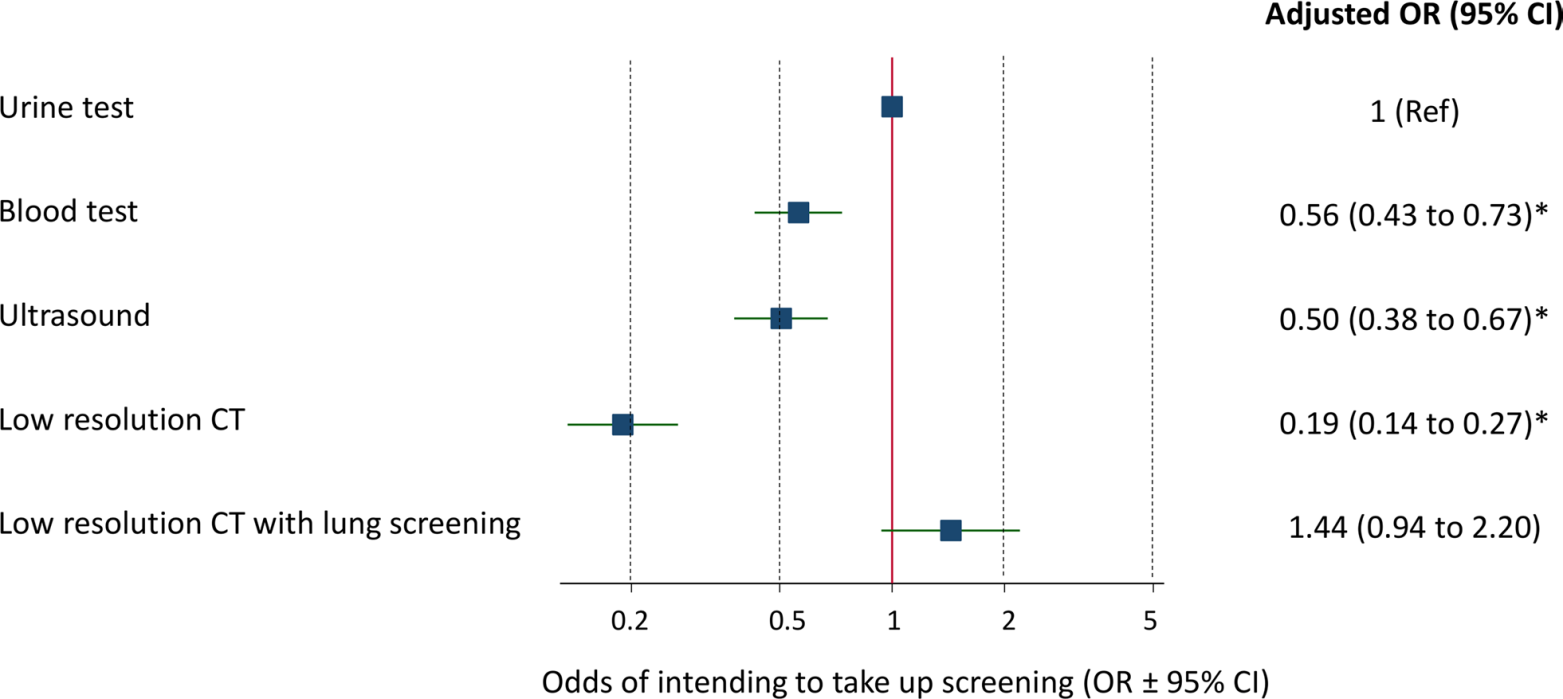
Odds (OR ± 95% confidence intervals) of intending to take up screening with each of the five screening modalities, adjusting for participant characteristics, the burden/inconvenience and worry associated with each test, and clustering of response by participant. *significantly lower than urine test (p<0.05)

After adjusting for participant characteristics (age, sex, education level, ethnicity, self-reported general health, country of residence, income group, BMI, smoking status, prior history of cancer, family history of kidney cancer), cancer worry, perceived risk of cancer, beliefs about cancer outcomes and treatments, and the burden/inconvenience and worry associated with the tests, and clustering of response by participant, there were significant differences between the different tests in intention to take up screening (Fig. 2). In particular, compared with intention to take up screening with a urine test, participants were half as likely to report that they intended to undergo blood [OR 0.56 (0.43–0.73)] or ultrasound [OR 0.50 (0.38–0.67)] testing compared to urine testing, and half again as likely to report that they intended to take part in a screening programme featuring a low dose CT scan [OR 0.19 (0.14–0.27)).

**Fig. 3:**
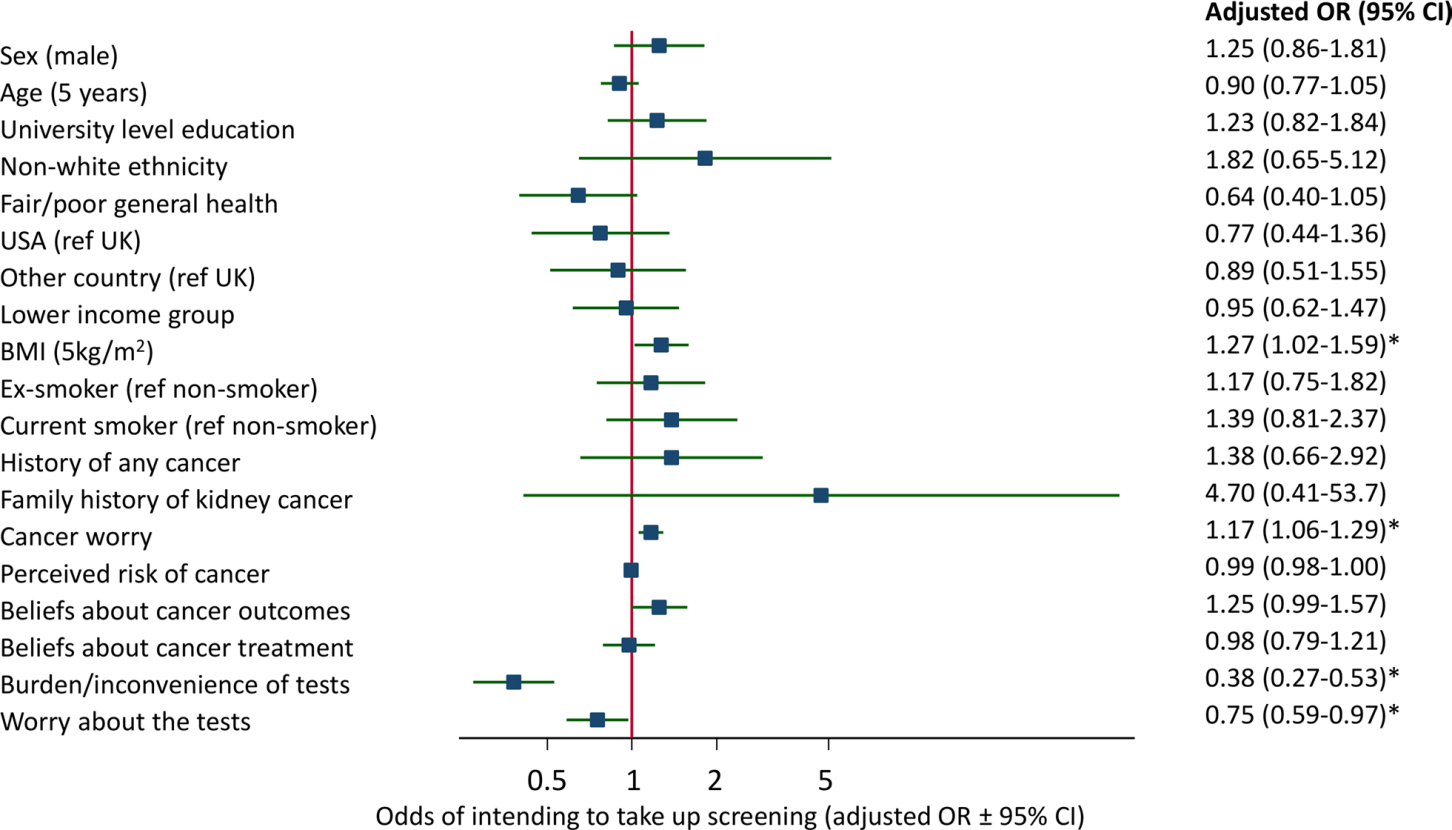
The association between participant characteristics and intention to attend screening across all screening modalities. Odds ratios (OR) are adjusted for all factors in the figure. Cancer worry is measured on a scale from 3 to 15, perceived risk of cancer from 0 to 100, beliefs about cancer outcomes from 1.3 to 6.6, beliefs about cancer treatment from 1 to 5, burden/inconvenience and worry about the tests from 1 to 5.* *p* < 0.05

Participants with higher BMI and higher general cancer worry were more likely to report intention to attend screening (Fig. 3). Being more worried about the screening tests or associating more burden and inconvenience were associated with decreased odds of intending to take up screening overall in the study population. There was, however, an interaction between general cancer worry and worry about the screening tests, with worry about screening tests associated with reduced odds of intending to attend only in those with low cancer worry (cancer worry scale ≤ 5) (OR 0.67 (0.48–0.94)) and not in than those with high cancer worry (OR 0.98 (0.69–1.40)). There was no evidence of an interaction (p>0.05) for the other potential interactions assessed. Although not statistically significant as a result of the small numbers with a family history of kidney cancer, having a family history of kidney was also associated with an increased odds of intending to take up screening (OR 4.70 (0.41–53.7), with 27 of the 29 participants with a family history of kidney cancer likely to take up all four screening tests. These findings were similar when each screening option was considered separately (Additional file 3: Supplementary Table 2 and Additional file 4: Supplementary Fig. 1a-e). Notably all those with a family history of kidney cancer reported they would take up screening with an ultrasound scan, 28 out of 29 would take up screening with urine or blood testing, and 27 out of 29 would take up low-dose CT scanning.

The reported influence of other factors on intention to attend kidney cancer screening are reported in Table 4. Having symptoms of kidney cancer and a recommendation by their GP were the most influential factors.

**Table 4.** Factors influencing intention to take up screening

	Influence on intention to take up screening		
	Much/slightly less likely (n, %)	No influence (n, %)	Much/slightly more likely (n, %)
If it was recommended by my GP?	11 (1.1)	129 (12.6)	881 (86.3)
If I had symptoms of kidney cancer?	13 (1.3)	45 (4.4)	963 (94.3)
If I could do the test at home?	26 (2.5)	277 (27.1)	718 (70.3)
If I could do the test at the GP?	20 (2)	278 (27.2)	723 (70.8)
If I had to go to the hospital for the test?	244 (23.9)	377 (36.9)	400 (39.2)
If I could make an appointment at the weekend or in the evenings?	28 (2.7)	465 (45.5)	528 (51.7)
If I had to leave work early?	179 (17.5)	657 (64.3)	185 (18.1)
If I could book the appointment online?	19 (1.9)	449 (44)	553 (54.2)

## Discussion

To the best of our knowledge, this study is the first to assess public attitudes towards potential kidney cancer screening programmes. Participants expressed high levels of intention to take-up kidney cancer screening, both within kidney cancer specific screening programmes and in conjunction with lung cancer screening. This was despite over 80% knowing nothing about kidney cancer or having only heard of the condition before participating in the survey. There were significant differences in intention between screening modalities, with a preference for urine testing or low-dose CT combined with lung cancer screening over a blood test, ultrasound scan or low-dose CT alone. Participants were also more likely to intend to undergo screening if they reported higher general cancer worry or less burden/inconvenience associated with the screening test, and if they had symptoms of kidney cancer or their GP had recommended screening. Less worry about the screening test was also associated with higher intention to attend, but only in those with lower general cancer worry. For those with higher general cancer worry, the general worry about cancer appeared to dominate any worry associated with the screening test itself.

### Comparison with literature

The overall positive attitude towards a screening programme for kidney cancer mirrors the general enthusiasm for cancer screening seen in population based surveys [20] and findings of a similar study on a potential lung cancer screening programme [13]. In that study over 90% of survey respondents believed that there was benefit to lung cancer screening, with the majority agreeing that lung screening could reduce chances of lung cancer death and only a small minority endorsing avoidance of lung cancer screening due to fear of what might be found or low perceived effectiveness of screening.

The observed differences between tests in intention to take part in a screening programme, with a preference for urine testing over a blood test, ultrasound scan or low-dose CT alone, is also consistent with existing literature which reports a preference for non-invasive tests requiring no preparation and causing no pain or long-term harm [21–23]. The greater intention seen for low-dose CT in combination with lung cancer screening likely reflects greater acceptability for combination screening, as seen in the context of a ‘One Stop’ cancer screening programme [24]. Given the low prevalence of kidney cancer, such a ‘bolt on’ screening programme may well be the most cost-effective as well as the most acceptable. It may also increase uptake of existing programmes [25].

### Strengths and limitations

The survey was developed with the support of patient and public representatives in order to maximise participants’ understanding and, where possible, previously validated questions were used. The main limitation is our use of an online recruitment platform. Although the platform we used has been developed specifically for research and enabled us to rapidly recruit participants of different ages and from different socio-economic backgrounds, compared with the UK and US population, the participants in this study were more likely to have university level education (46% in this study compared with 40% in the UK [26] and 35% in the US [27]) and be of White ethnicity (95% in this study compared with 86% in the UK [28] and 76% in the US [29]) and high socioeconomic status (76% in social group ABC1 in this study compared with 54% in England [30]). Uptake of other cancer screening programmes is known to be around 5–15% higher amongst these groups than those without university education, of ethnic minority groups and in lower social classes [31, 32]. The high proportion of participants expressing an intention to attend screening observed in this study may therefore be an over-estimation of intention in the wider population. Additionally, Prolific members are experienced in completing online tasks and their views may not be representative of those of the general population [33]. However, while these differences might lead to over-estimation of levels of intention and affect the absolute levels of worry and burden reported by the respondents as a whole, the differences observed between screening tests and the associations between worry and burden and other participant socio-demographic characteristics and intention to take up screening would be expected to be generalizable to other populations [34]. In the absence of an existing screening programme we were also only able to measure intention and not attendance at screening. Although there is an established relationship between screening intention and screening attendance [35], there is known to be an ‘intention-behaviour gap’ which is modified by various factors [36, 37]. The actual uptake of any future screening programme is therefore likely to be lower than the estimates of intention in this study. The uptake of any future screening programme will also likely depend on the information provided to eligible individuals. As data are currently not available on all the potential benefits and harms of kidney cancer screening, the information provided to participants in this study was based on estimates from the best available evidence and provided in less detail that would likely be included in any future screening programme. In particular, although we mentioned the possibility of both false positive and false negative findings as a result of screening, we did not provide participants with specific details on the possibility of detecting benign small renal masses or data on the growth rate of small incidentally detected tumours. With on-going research in this area, especially surrounding potential blood and urine biomarkers and approaches to the management of small renal masses, these estimates may change, making screening either more or less favourable to individuals.

## Conclusion

Among the participants in our study, we found that there is an overall enthusiasm for kidney cancer screening, with high potential uptake rates. Our findings also highlight the influence of screening modality, with particular support for urine and low-dose CT combined with lung cancer screening, as well as individual-level and system-level factors that are likely to influence uptake. Together these findings support on-going research into kidney cancer screening tests and the potential for combining kidney cancer screening with existing or new screening programmes.

## Supplementary information

**Additional file 1**. Participant invitation email.

**Additional file 2**. Exploring attitudes towards kidney cancer screening survey.

**Additional file 3**. Supplementary Tables.

**Additional file 4**. Supplementary Figures.

## Data Availability

The dataset supporting the conclusions of this article will be made available in the University of Cambridge data repository (https://www.repository.cam.ac.uk/) for at least 10 years from the last access. All the data will be stored in accordance with the Data Protection Act 1998.

## Abbreviations

BMI: Body mass index
CT: Computed tomography
OR: Odds ratio
QALY: Quality adjusted life year
SD: Standard deviation
SES: Socioeconomic status
USS: Ultrasound scan
