# Supplementary material for: Public attitudes towards screening for kidney cancer: an online survey
Source: BMC Urol. 2020 Oct 28;20:170. doi: 10.1186/s12894-020-00724-0 (PMC7592501; doi:10.1186/s12894-020-00724-0)
Supplement: Supplementary file 1 — Additional file 1. Participant invitation email. [file 12894_2020_724_MOESM1_ESM.pdf]

Prolific

New study available!

Hi NAME,

Jim from Prolific here. We've got a study running right now **"Exploring people's attitudes towards kidney cancer screening"** that needs some more participants, and you're eligible!

We'd love it if you could help out the researcher Juliet Usher-Smith by taking part, and at the same time you can earn a reward of £2.50 for 25 minutes of your time.

Participate Now

Best,

Jim

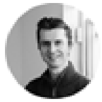

© 2017 Prolific
