# Supplementary material for: Public attitudes towards screening for kidney cancer: an online survey
Source: BMC Urol. 2020 Oct 28;20:170. doi: 10.1186/s12894-020-00724-0 (PMC7592501; doi:10.1186/s12894-020-00724-0)
Supplement: Supplementary file 2 — Additional file 2. Exploring attitudes towards kidney cancer screening survey. [file 12894_2020_724_MOESM2_ESM.docx]

**Supplementary File 2 - Exploring attitudes towards kidney cancer screening survey**

| Question | Answer | Validation of question/reference |
| --- | --- | --- |
| *[Note that this content was delivered online so the formatting looked slightly different to in this document. The participants also did not see the headings in grey boxes or the references, but these are included to illustrate where the questions have come from.]* | | |
| Thank you for agreeing to complete this questionnaire. Please answer every question. If you are uncertain about how to answer a question, then please select the closest option. | | |
| ***Demographic information***  *In this first section we would like to ask you a few questions about yourself. These questions allow us to make sure we are including people from a range of different backgrounds and see if different groups of people have different views. We will not be able to identify you from your answers.* | | |
| How old are you? | - 45-49 - 50-54 - 55-59 - 60-64 - 65-69 - 70-74 - 75-79 |  |
| What is your ethnic group? Choose one option that best describes your ethnic group or background. | - White - Mixed/Multiple ethnic group - Asian/Asian British - Black/African/Caribbean/Black British - Other, please describe______ |  |
| What is your highest education level? | - Finished school at or before the age of fifteen - Completed CSEs, O-levels or equivalent - Completed A Levels or equivalent - Completed further education but not a degree - Completed a Bachelor’s degree / Masters’ degree / PHD - Other (please specify) ______ | Validated from Cancer Awareness Measure (CAM) toolkit, (pre-2014, as 2014 onwards has not been validated)^2^ |
| Are you currently: | - Employed full-time - Employed part-time - Unemployed - Self-employed - Full-time homemaker - Retired - Still studying - Disabled or too ill to work - Prefer not to say | Validated from Cancer Awareness Measure (CAM) toolkit, (pre-2014, as 2014 onwards has not been validated)^2^ |
| Please indicate to which occupational group the Chief Income Earner in your household belongs, or which group fits best. This could be you: the Chief Income Earner is the person in your household with the largest income. If the Chief Income Earner is retired and has a pension please answer for their most recent occupation. If the Chief Income Earner is not in paid employment but has been out of work for less than 6 months, please answer for their most recent occupation. | - Professional or higher technical work - work that requires at least degree-level qualifications (e.g. doctor, accountant, schoolteacher, university lecturer, social worker, systems analyst) - Manager or Senior Administrator (e.g. company director, finance manager, personnel manager, senior sales manager, senior local government officer) - Clerical (e.g. clerk, secretary) - Sales or Services (e.g. commercial traveller, shop assistant, nursery nurse, care assistant, paramedic) - Foreman or Supervisor of Other Workers (e.g building site foreman, supervisor of cleaning workers) - Skilled Manual Work (e.g. plumber, electrician, fitter, train driver, cook, hairdresser) - Semi-Skilled or Unskilled Manual Work (e.g. machine operator, assembler, postman, waitress, cleaner, labourer, driver, bar-worker, call centre worker) - Other (Please specify) - Have never worked | Validated method of determining social class from National Readership Survey, used by Smits et al.,2018 to give ABC1 (higher SES) and C2DE (lower SES) ^23,4^ |
| **Lifestyle and screening history** | | |
| ***Lifestyle and screening history***  *The following questions are about your lifestyle and your past screening decisions. Please answer as honestly as you can as this will help us with our analysis.* | | |
| In general, would you say your health is | - Excellent - Very good - Good - Fair - Poor | Validated question from CDC for self-rate health^5^. |
| What is your smoking status? | - Never smoked - Used to smoke - Smoke up to 20 per day - Smoke 20 or more per day | From lung cancer study, Smits et al., 2018^3^ |
| What is your height? | - ____ |  |
| What is your weight? | - ____ |  |
| Have you been invited to take part in any of the following screening programmes (please note that they are not offered to everyone)?  Information is in the format of (what it involves, who it is offered to) | - Yes/No/Not Eligible/Don’t know   - Abdominal Aortic Aneurysm screening (ultrasound, men aged over 65)   - Bowel Cancer screening (poo sample or colonoscopy, men and women aged 60-74)   - Breast Cancer screening (mammogram, women aged 50-70)   - Cervical Cancer screening (smear test, women aged 25-64) |  |
| Which of the following screening programmes did you take up the invitation to take part in? | - Yes/No/I wasn’t invited/Don’t know   - Abdominal Aortic Aneurysm screening (ultrasound, men aged over 65)   - Bowel Cancer screening (poo sample or colonoscopy, men and women aged 60-74)   - Breast Cancer screening (mammogram, women aged 50-70)   - Cervical Cancer screening (smear test, women aged 25-64) |  |
| **Thoughts and beliefs about cancer in general** | | |
| ***Thoughts and beliefs about cancer***  *The next section asks about your thoughts and beliefs about cancer in general. Please answer as honestly as you can as this will help us with our analysis. The questions are about YOUR opinion.* | | |
| Have you ever had cancer? | - Yes - No |  |
| For each of the following statements please select the option that best applies to you:   - During the past month, how often have you thought about your own chances of getting cancer? - During the past month, how often have thoughts about your chances of getting cancer affected your mood? - During the past month, how often have thoughts about your chances of getting cancer affected your ability to perform your daily activities? | - Not at all (1) - Rarely - Sometimes - Often - A lot (5) | Validated questions from Lerman cancer worry scale^6^ |
| How likely do you think is it that you will get cancer at some point in the next 10 years? | - Unlikely (0) - Likely (100) |  |
| Below are some statements that are sometimes made about cancer. For each of these statements how much do you agree or disagree with them?   1. These days, many people with cancer can expect to continue with normal activities and responsibilities 2. Most cancer treatment is worse than the cancer itself 3. I would NOT want to know if I have cancer 4. Cancer can often be cured 5. Going to the doctor as quickly as possible after noticing a symptom of cancer could increase the chances of surviving. 6. Some people think a diagnosis of cancer is a death sentence. To what extent do you agree or disagree that a diagnosis of cancer is a death sentence? | - Strongly agree (1) - Agree - Neither disagree nor agree - Disagree - Strongly disagree (5) | Kidney cancer **beliefs**, taken from Smits et al., 2018^3^ (and made specific to kidney cancer, rather than lung cancer), which were based on validated questions from the ABC measure^7^.  Questions 1, 4 and 6 were used as an aggregate measure of ‘beliefs about cancer outcomes’, and combined using published co-efficients^7^. |
| It is important that you pay attention to this study. Please select “Strongly Disagree” | - Strongly agree - Agree - Neither disagree nor agree - Disagree - Strongly disagree |  |
| **Kidney cancer awareness** | | |
| ***Kidney cancer awareness***  *This section is about your awareness of kidney cancer specifically; it is not assessing your personal risk of cancer. This is not a test, we are interested in your thoughts and beliefs so please answer the questions as honestly as you can. It is important that you do not use a search engine to look up any answers, as we are interested in YOUR opinion.* | | |
| Have you ever had kidney cancer? | - Yes - No |  |
| Have your parents or any brothers or sisters ever had kidney cancer? | - Yes - No - Don’t know/prefer not to answer |  |
| Outside your immediate family, do you know anyone who has had kidney cancer? | - Yes - No |  |
| How much do you know about kidney cancer? | - Nothing at all - I have only heard of the term before - I know a little about the disease - I am very familiar with it | Taken from ‘Exploring Knowledge, Attitudes, and Practices (KAP) Related to Breast and Cervical Cancers in Mongolia’ and made relevant to kidney cancer. This is not a validated question, but was developed using literature and other KAP surveys^1^ |
| The following may or may not be warning signs for kidney cancer.  Do you think ____ could be a sign of kidney cancer?   - **Blood in your urine** - **A persistent pain in your lower back or side, just below your ribs** - Coughing up blood - **A lump or swelling in your side** - **Extreme tiredness (fatigue)** - Difficulty swallowing - Blood in the stool - **Loss of appetite and weight loss** - **Persistent high blood pressure** - **Night sweats** - **In men, swelling of the veins in the testicles** | - Yes (1) - No (-1) - Don’t know (0) | Validated questions from CAM toolkit for bowel cancer Question 2, but the answers for warning signs (**in bold**) have been replaced with symptoms from the NHS website for kidney ^8,9^  A score for symptom awareness was calculated using a score of 1 for correct answers, -1 for incorrect answers and 0 for ‘Don’t know’ which were summed for each participant. Participants were then categorised into two groups (high/low) based on whether their overall score was above or below the median score. |
| These are some of the things that can increase a person’s chance of developing cancer. How much do you agree that each of these can increase a person’s risk of developing kidney cancer?   - **Smoking any cigarettes at all** - **Exposure to another person’s cigarettes** - Drinking more than 1 unit of alcohol a day - Eating less than 5 portions of fruit and vegetables a day - Eating red or processed meat once a day or more - **Being overweight (Body Mass Index (BMI) over 25)** - Getting sunburnt more than once as a child - **Being over 70 years old** - **Having a close relative with kidney cancer** - Infection with HPV (Human Papillomavirus) - Doing less than 30 minutes of moderate physical activity 5 times per week - **Diabetes** - **High blood pressure** | - Strongly disagree (1) - Disagree - Not sure - Agree - Strongly agree (5) | Validated questions from CAM cancer toolkit^2^ where risk factors for kidney cancer have been included (**in bold**) based on literature, as well as risk factors for other cancer^10^  A score for risk factor awareness was calculated by giving responses to the five point Likert scale (strongly disagree to strongly agree) a score of 1 to 5 for those risk factors associated with kidney cancer and a reverse score from 5 to 1 for those not associated with kidney cancer. These were summed to give a total for each participant. Participants were then categorised into two groups (high/low) based on whether their overall score was above or below the median score. |
| **Kidney cancer screening questions - Information given about kidney cancer potential screening tests** | | |
| ***Kidney cancer screening tests***  *The following section is about screening for kidney cancer. You will be shown information about kidney cancer and potential screening tests and then asked your views on different screening options.*  *Kidney cancer is the 7th most common cancer in the UK. There are around 12,600 new kidney cancer cases in the UK every year and 6000 deaths from kidney cancer. In about half of the cases of kidney cancer, there are no obvious symptoms at first. These cases are often picked up during tests carried out for other reasons. Kidney cancer can often be cured, normally by surgery, if it is caught early. However, if it is not diagnosed until it has spread beyond the kidney, then a cure is much less likely. In the UK at the moment nearly a third of all cases of kidney cancer are diagnosed after the kidney cancer had spread beyond the kidneys.*  *Currently there is no screening programme for kidney cancer. There is potential for a screening programme to be developed in the future though. This may help detect kidney cancer at an early stage, making it easier to treat and reducing the number of people who die from kidney cancer. As with other screening programmes, it would involve having screening tests. If these tests find anything unusual, further tests (usually a detailed CT scan) are needed to confirm a possible kidney cancer. If they find a lump in the kidney then taking a small sample of that may be recommended to confirm or rule out cancer. Some patients wish to have surgery to remove the lump without confirming it is cancer and some of these lumps will not be cancer. As such some people may have an operation that is not required and others will be worried that they have cancer when they don’t. There is also a chance that a cancer could be missed as no screening test is 100% reliable. Some people will therefore be told that they don’t have cancer when they do.*  *Over the next few pages you will see some information about each of the potential screening tests. You will then be asked how much inconvenience, burden or worry you associate with each and how likely it is that you would attend for screening with each if you were invited. Please read through the information about the tests carefully so that you can use that information to help you answer those questions.* | | |
| ***Ultrasound scan***   - *Ultrasound scanning can be used to scan the kidneys. It is similar to the scan offered to pregnant women* - *Ultrasound scans use high frequency sound waves to make a picture of organs inside your body.* - *You would need to go to a clinic or hospital for the scan. At the scan you would be asked to lie down and show your abdomen (tummy) by lifting your top. You would not need to undress. A cool gel would be put on your skin.* - *The ultrasound sensor would be moved over your skin on your lower back, in order to make a picture of each kidney which will appear on a screen.* - *Ultrasound scans are not painful and there are no risks from the scans themselves. They do not involve exposure to radiation. Most ultrasound scans last between 15 and 45 minutes* - *If all adults in the UK were offered ultrasound screening, around 3-4 out of every 1000 people screened would have an abnormal scan and require further investigations. Approximately half of those will have kidney cancer.* | | |
| ***Low-dose computed tomography (CT) scan***   - *A low-dose computed tomography (CT) scan uses x-rays to make a detailed 3 dimensional (3D) picture of the kidneys. You would need to go to a clinic or hospital for the scan. At the scan you would lie on the machine couch on your back, and the couch slowly moves through the hole of the scanner. The scanner doesn't surround your whole body at once, so you shouldn't feel claustrophobic.* - *This type of CT scan uses no dyes, no injections, and requires nothing to swallow by mouth* - *The actual scan takes 1-2 minutes, and the appointment would normally take around 15 minutes.* - *CT scans are quick, painless and generally safe.* - *You would be exposed to X-ray radiation during the scan. Generally, the amount of radiation you're exposed to during each scan is the equivalent 6 months of exposure to natural radiation from the environment. It's thought exposure to radiation during CT scans could slightly increase your chances of developing cancer many years later, although this risk is thought to be very small (less than 1 in 2,000).* - *If all people in the UK were offered screening with CT, around 3-4 out of every 1000 people screened would have an abnormal scan and require further investigations. Approximately half of those will have kidney cancer.* | | |
| ***Urine sample***   - *In the future we hope to be able to test for biomarkers in the urine to identify people who are likely to have kidney cancer.* - *This approach doesn’t diagnose you with kidney cancer, but it is a simple way to find out if you need further tests.* - *This would require you to provide a urine sample in a pot and either send it off or give to your GP. Your urine sample will then be tested to see if the biomarkers are present.* - *There are no complications from the test itself but if a test is positive for the kidney cancer biomarkers you may have to have further tests.* - *If all people in the UK were offered screening with a urine sample, estimates are that around 2-3 out of every 1000 people screened would have an abnormal test and require further investigations. Most of those will have kidney cancer.* | | |
| ***Blood test***   - *In the future we hope to be able to test for biomarkers in the blood to identify people who are likely to have kidney cancer.* - *This approach doesn’t diagnose you with kidney cancer, but it is a simple way to find out if you need further tests.* - *This would require you to have a blood test at your GP’s surgery. The blood sample will tested at a laboratory to see if the biomarkers are present.* - *A blood test can be slightly painful and leave a bruise.* - *If the test is positive for the kidney cancer biomarkers you may have to have further tests.* - *If all people in the UK were offered screening with a blood test, around 2-3 out of every 1000 people screened would have an abnormal test and require further investigations. Most of those will have kidney cancer.* | | |
| Based on the information you have just read, how much inconvenience or burden do you associate with having an **ultrasound scan**? | - No inconvenience or burden (1) - Very great inconvenience or burden (5) |  |
| Based on the information you have just read, how much inconvenience or burden do you associate with having a **low dose CT scan?** | - No inconvenience or burden (1) - Very great inconvenience or burden (5) |  |
| Based on the information you have just read, how much inconvenience or burden do you associate with having a **urine test**? | - No inconvenience or burden (1) - Very great inconvenience or burden (5) |  |
| Based on the information you have just read, how much inconvenience or burden do you associate with having a **blood test**? | - No inconvenience or burden (1) - Very great inconvenience or burden (5) |  |
| If you were invited to have an **ultrasound scan** as part of a screening programme for kidney cancer, how worried would you be about having the scan? | - Not at all worried (1) - Extremely worried (5) |  |
| If you were invited to have an **low dose CT scan** as part of a screening programme for kidney cancer, how worried would you be about having the scan? | - Not at all worried (1) - Extremely worried (5) |  |
| If you were invited to have an **urine test** as part of a screening programme for kidney cancer, how worried would you be about having the test? | - Not at all worried (1) - Extremely worried (5) |  |
| If you were invited to have an **blood test** as part of a screening programme for kidney cancer, how worried would you be about having the test? | - Not at all worried (1) - Extremely worried (5) |  |
| If there was an **ultrasound based screening programme** and you were invited to have an **ultrasound scan** as part of that screening programme for kidney cancer, how likely do you think it is that you would choose to take part in the screening? | - Very likely - Likely - Unlikely - Very unlikely |  |
| Please describe in a few words why? | - Free text:_____ |  |
| If there was a **low-dose CT based screening programme** and you were invited to have a **low-dose** **CT scan** as part of a screening programme for kidney cancer, how likely do you think it is that you would choose to take part in the screening? | - Very likely - Likely - Unlikely - Very unlikely |  |
| Please describe in a few words why? | - Free text:_____ |  |
| If there was a **urine sample based screening programme** and you were invited to give a **urine sample** as part of a screening programme for kidney cancer, how likely do you think it is that you would choose to take part in the screening? | - Very likely - Likely - Unlikely - Very unlikely |  |
| Please describe in a few words why? | - Free text:_____ |  |
| If there was a **blood sample based screening programme** and you were invited to give a **blood sample** as part of a screening programme for kidney cancer, how likely do you think it is that you would choose to take part in the screening? | - Very likely - Likely - Unlikely - Very unlikely |  |
| Please describe in a few words why? | - Free text:_____ |  |
| **Kidney cancer attitudes and barriers/facilitators for screening** | | |
| ***Attitudes towards kidney cancer screening***  *The following questions are about your attitudes towards screening. The invitation to take up screening refers to which ever type of screening is selected by the experts developing the programme.* | | |
| - I would be so worried about what might be found at kidney cancer screening that I would prefer not to have it. - Kidney cancer screening is only necessary if I have symptoms - I don’t think there is any point going for kidney cancer screening because it won’t affect the outcome - Kidney cancer screening could reduce my chance of dying from kidney cancer. | - Strongly agree - Agree - Neither disagree nor agree - Disagree - Strongly disagree | Kidney cancer screening **attitudes**, taken from Smits et al., 2018^3^ (and made specific to kidney cancer, rather than lung cancer) which were based on validated questions from the ABC measure^7^. These were the only questions in the ABC survey that were directly about screening. |
| If you were invited for kidney cancer screening, how would each of the following influence your decision whether to take up the invitation?   - If it was recommended by my GP? - If I had symptoms of kidney cancer? - If I could do the test at home? - If I could do the test at the GP? - If I had to go to the hospital for the test? - If I could make an appointment at the weekend or in the evenings? - If I had to leave work early? - If I could book the appointment online? | - Much less likely to attend - Slightly less likely to attend - No influence - Slightly more likely to attend - Much more likely to attend | Barriers and facilitators are based on literature (not validated)   - Ali et al 2015 Self-reported reasons for non-uptake of high-risk lung cancer screening^11^ |
| **Kidney cancer screening combined with lung cancer screening** | | |
| A number of studies are currently testing a new lung cancer screening programme in which people who are at high risk of developing lung cancer (based on their **age and smoking history**) are invited for low-dose CT scans. At the moment these scans only go down as far as the top of the kidney. There may be the option to extend those scans to include the whole of the kidney. Those individuals could then choose whether to also have screening for kidney cancer at the same time. The risk factors for lung cancer and kidney cancer are similar but not exactly the same.  The advantages of this approach are that it would be very cost effective and time effective for participants. The disadvantages are that some people may be at high risk of kidney cancer but not lung cancer and so would not be invited.   - How reasonable do you think it would be to offer kidney cancer screening with low-dose CT to people having lung cancer screening? - How reasonable do you think it would be to ***only*** offer kidney cancer screening with low-dose CT to people having lung cancer screening? i.e. people not at high risk of lung cancer would not be invited. | - Not at all reasonable – extremely reasonable (0-6) | Not validated |
| If you were invited for lung cancer screening and given the option to also have kidney cancer screening at the same time, how likely do you think it is that you would choose to have the kidney cancer screening? | - Very likely - Likely - Unlikely - Very unlikely |  |
| If you were invited for lung cancer screening, how would being invited to also have kidney cancer screening influence your decision whether to take up the invitation for lung cancer screening? | - Much less likely to attend - Slightly less likely to attend - No influence - Slightly more likely to attend - Much more likely to attend |  |
| *Thank you for completing the survey. Your time and responses are very much appreciated.* | | |

References

1 Yerramilli P, Dugee O, Enkhtuya P, *et al.* Exploring Knowledge, Attitudes, and Practices Related to Breast and Cervical Cancers in Mongolia: A National Population-Based Survey. *Oncologist* 2015;**20**:1266–73. doi:10.1634/theoncologist.2015-0119

2 Cancer Awareness Measure toolkit version 2.1 Cancer Research UK Cancer Awareness Measure (CAM) Toolkit (version 2.1). 2007. https://www.cancerresearchuk.org/sites/default/files/health_professional_cancer_awareness_measure_toolkit_version_2.1_09.02.11.pdf (accessed 28 May 2019).

3 Smits SE, McCutchan GM, Hanson JA, *et al.* Attitudes towards lung cancer screening in a population sample. *Heal Expect* 2018;**21**:1150–8. doi:10.1111/hex.12819

4 Social Grade | National Readership Survey. http://www.nrs.co.uk/nrs-print/lifestyle-and-classification-data/social-grade/ (accessed 30 May 2019).

5 2006 Behavioral Risk Factor Surveillance System Questionnaire. 2006. https://www.cdc.gov/brfss/questionnaires/pdf-ques/2006brfss.pdf (accessed 3 Jun 2019).

6 Lerman C, Trock B, Rimer BK, *et al.* Psychological side effects of breast cancer screening. *Health Psychol* 1991;**10**:259–67.

7 Simon AE, Forbes LJL, Boniface D, *et al.* An international measure of awareness and beliefs about cancer: development and testing of the ABC. *BMJ Open* 2012;**2**. doi:10.1136/bmjopen-2012-001758

8 Bowel cancer Awareness Measure toolkit Version 2.1 Cancer Research UK Bowel Cancer Awareness Measure (CAM) Toolkit. 11AD. www.data-archive.ac.uk (accessed 30 May 2019).

9 Kidney cancer - Symptoms - NHS. https://www.nhs.uk/conditions/kidney-cancer/symptoms/ (accessed 30 May 2019).

10 Chow W-H, Dong LM, Devesa SS. Epidemiology and risk factors for kidney cancer. *Nat Rev Urol* 2010;**7**:245–57. doi:10.1038/nrurol.2010.46

11 Ali N, Lifford KJ, Carter B, *et al.* Barriers to uptake among high-risk individuals declining participation in lung cancer screening: a mixed methods analysis of the UK Lung Cancer Screening (UKLS) trial. *BMJ Open* 2015;**5**:e008254. doi:10.1136/bmjopen-2015-008254

12 Hunt JD, van der Hel OL, McMillan GP, *et al.* Renal cell carcinoma in relation to cigarette smoking: Meta-analysis of 24 studies. *Int J Cancer* 2005;**114**:101–8. doi:10.1002/ijc.20618

13 Piper MS, Maratt JK, Zikmund-Fisher BJ, *et al.* Patient Attitudes Toward Individualized Recommendations to Stop Low-Value Colorectal Cancer Screening. *JAMA Netw Open* 2018;**1**:e185461. doi:10.1001/jamanetworkopen.2018.5461
