# Supplementary material for: Public attitudes towards screening for kidney cancer: an online survey
Source: BMC Urol. 2020 Oct 28;20:170. doi: 10.1186/s12894-020-00724-0 (PMC7592501; doi:10.1186/s12894-020-00724-0)
Supplement: Supplementary file 3 — Additional file 3. Supplementary Tables. [file 12894_2020_724_MOESM3_ESM.docx]

Supplementary Table 1. Awareness of risk factors and symptoms for kidney cancer

| **Risk factor** | **Strongly agree**  **(n, %)** | **Agree**  **(n, %)** | **Not sure**  **(n, %)** | **Disagree**  **(n, %)** | **Strongly disagree (n, %)** |
| --- | --- | --- | --- | --- | --- |
| Smoking any cigarettes at all* | 10 (1.0) | 34 (3.3) | 228 (22.3) | 450 (44.1) | 299 (29.3) |
| Exposure to another person’s cigarettes* | 12 (1.1) | 87 (8.5) | 325 (31.8) | 480 (47.0) | 117 (11.5) |
| Being overweight (Body Mass Index (BMI) over 25)* | 3 (0.3) | 42 (4.1) | 230 (22.5) | 591 (57.9) | 155 (15.1) |
| Being over 70 years old* | 14 (1.4) | 112 (11.0) | 352 (34.5) | 500 (49.0) | 43 (4.2) |
| Having a close relative with kidney cancer* | 10 (1.0) | 57 (5.6) | 232 (22.7) | 587 (57.5) | 135 (13.2) |
| Diabetes* | 7 (0.7) | 86 (8.4) | 464 (45.4) | 403 (39.5) | 61 (6.0) |
| High blood pressure* | 9 (0.9) | 109 (10.7) | 560 (54.8) | 304 (29.8) | 39 (3.8) |
| Drinking more than 1 unit of alcohol a day | 118 (11.6) | 481 (47.1) | 318 (31.1) | 94 (9.2) | 10 (1.0) |
| Eating less than 5 portions of fruit and vegetables a day | 32 (3.1) | 262 (25.7) | 409 (40.1) | 283 (27.7) | 35 (3.4) |
| Eating red or processed meat once a day or more | 62 (6.1) | 355 (34.8) | 435 (42.6) | 149 (14.6) | 20 (2.0) |
| Getting sunburnt more than once as a child | 15 (1.5) | 61 (6.0) | 303 (29.7) | 495 (48.5) | 147 (14.4) |
| Infection with HPV (Human Papillomavirus) | 21 (2.1) | 165 (16.2) | 679 (66.5) | 135 (13.2) | 21 (2.1) |
| Doing less than 30 minutes of moderate physical activity 5 times per week | 16 (1.6) | 282 (27.6) | 415 (40.6) | 273 (26.7) | 35 (3.4) |
| **Symptom** | **Correct**  **(n, %)** | | **Not sure**  **(n, %)** | **Incorrect**  **(n, %)** | |
| Blood in your urine* | 917 (89.8) | | 85 (8.3) | 19 (1.9) | |
| A persistent pain in your lower back or side, just below your ribs* | 838 (82.1) | | 162 (15.9) | 21 (2.1) | |
| A lump or a swelling in your side* | 623 (61.0) | | 313 (30.7) | 85 (8.3) | |
| Extreme tiredness (fatigue)* | 776 (76.0) | | 206 (20.2) | 39 (3.8) | |
| Loss of appetite and weight loss* | 829 (81.2) | | 158 (15.5) | 34 (3.3) | |
| Persistent high blood pressure* | 293 (28.7) | | 567 (55.5) | 161 (15.8) | |
| Night sweats* | 334 (32.7) | | 513 (50.2) | 174 (17.0) | |
| Coughing up blood | 418 (40.9) | | 355 (34.8) | 248 (24.3) | |
| Difficulty swallowing | 488 (47.8) | | 442 (43.3) | 91 (8.9) | |
| Blood in the stool | 320 (31.3) | | 292 (28.6) | 409 (40.1) | |
| In men, swelling of the veins in the testicles | 228 (22.3) | | 610 (59.8) | 183 (17.9) | |

* Risk factors and symptoms associated with kidney cancer where a ‘yes’ response was correct.

Supplementary Table 2. Univariable and multivariable associations with intention to likely or very likely take up screening with each of the tests

|  | **Urine** | | **Blood** | | **Ultrasound** | | **Low-dose CT** | | **Low-dose CT with lung** | |
| --- | --- | --- | --- | --- | --- | --- | --- | --- | --- | --- |
|  | **Univariable**  **(OR, 95% CI)** | **Multivariable**  **(OR, 95% CI)**  **n=756** | **Univariable**  **(OR, 95% CI)** | **Multivariable**  **(OR, 95% CI)**  **n=756** | **Univariable**  **(OR, 95% CI)** | **Multivariable**  **(OR, 95% CI)**  **n=732** | **Univariable**  **(OR, 95% CI)** | **Multivariable**  **(OR, 95% CI)**  **n=756** | **Univariable**  **(OR, 95% CI)** | **Multivariable**  **(OR, 95% CI)**  **n=704** |
| **Age (5 years)** | **0.83**  **(0.69-0.99)** | 0.86  (0.66-1.13) | 0.88  (0.76-1.02) | 0.92  (0.73-1.15) | **0.86**  **(0.74-0.99)** | 0.83  (0.67-1.01) | 1.06  (0.94-1.19) | 0.96  (0.82-1.14) | **0.71**  **(0.57-0.89)** | **0.66**  **(0.48-0.91)** |
| **Sex** |  |  |  |  |  |  |  |  |  |  |
| Female | 1 (Ref) | 1 (Ref) | 1 (Ref) | 1 (Ref) | 1 (Ref) | 1 (Ref) | 1 (Ref) | 1 (Ref) | 1 (Ref) | 1 (Ref) |
| Male | 0.92  (0.55-1.55) | 1.12  (0.52-2.40) | 0.85  (0.56-1.28) | 1.01  (0.55-1.86) | 1.14  (0.77-1.71) | 1.21  (0.68-2.14) | 1.21  (0.89-1.62) | 1.24  (0.80-1.93) | 1.14  (0.64-2.02) | 1.36  (0.56-3.28) |
| **University level education** | 1.40  (0.82-2.40) | 1.26  (0.58-2.72) | 1.08  (0.71-1.64) | 0.90  (0.48-1.67) | 1.26  (0.84-1.89) | 1.47  (0.81-2.65) | 1.10  (0.81-1.49) | 1.36  (0.86-2.15) | 1.51  (0.83-2.73) | 1.34  (0.56-3.21) |
| **Personal history of cancer** | 1.27  (0.38-4.16) | 2.11  (0.26-17.1) | 1.62  (0.58-4.57) | 2.30  (0.28-19.0) | 1.38  (0.54-3.53) | 1.55  (0.34-7.20) | 1.16  (0.61-2.21) | 1.27  (0.44-3.62) | 1.00  (0.30-3.32) | 1.26  (0.15-10.6) |
| **Family history of kidney cancer** | 1.69  (0.23-12.7) | 1.65  (0.18-15.6) | 2.91  (0.39-21.7) | 1.52  (0.18-12.9) | --- | --- | 3.70  (0.87-15.7) | 7.00  (0.72-68.6) | --- | --- |
| **General health** |  |  |  |  |  |  |  |  |  |  |
| Excellent, very good,  good | 1 (Ref) | 1 (Ref) | 1 (Ref) | 1 (Ref) | 1 (Ref) | 1 (Ref) | 1 (Ref) | 1 (Ref) | 1 (Ref) | 1 (Ref) |
| Fair, poor | **0.45**  **(0.26-0.79)** | **0.38**  **(0.17-0.85)** | 0.66  (0.41-1.06) | 0.55  (0.28-1.08) | **0.57**  **(0.36-0.88)** | 0.63  (0.32-1.21) | 0.86  (0.60-1.23) | 0.81  (0.47-1.40) | **0.47**  **(0.26-0.87)** | 0.52  (0.19-1.40) |
| **Ethnicity** |  |  |  |  |  |  |  |  |  |  |
| White | 1 (Ref) | 1 (Ref) | 1 (Ref) | 1 (Ref) | 1 (Ref) | 1 (Ref) | 1 (Ref) | 1 (Ref) | 1 (Ref) | 1 (Ref) |
| Non-white | 1.40  (0.33-5.92) | 1.19  (0.14-10.0) | 1.15  (0.40-3.28) | 0.92  (0.17-4.87) | 1.74  (0.53-5.70) | 3.63  (0.42-31.1) | 1.88  (0.79-4.49) | 1.65  (0.47-5.80) | 0.72  (0.21-2.40) | --- |
| **Country** |  |  |  |  |  |  |  |  |  |  |
| UK | 1 (Ref) | 1 (Ref) | 1 (Ref) | 1 (Ref) | 1 (Ref) | 1 (Ref) | 1 (Ref) | 1 (Ref) | 1 (Ref) | 1 (Ref) |
| USA | 0.68  (0.36-1.26) | 1.03  (0.31-3.44) | 0.86  (0.51-1.45) | 1.61  (0.56-4.62) | **0.57**  **(0.36-0.92)** | 0.57  (0.25-1.33) | **0.61**  **(0.42-0.89)** | **0.47**  **(0.24-0.91)** | **0.45**  **(0.23-0.87)** | 1.12  (0.24-5.24) |
| Other | 2.09  (0.63-6.90) | 1.31  (0.34-5.09) | **2.87**  **(1.02-8.04)** | 2.47  (0.74-8.25) | 1.17  (0.57-2.43) | 0.67  (0.29-1.54) | 0.78  (0.48-1.27) | 0.78  (0.41-1.49) | 0.82  (0.31-2.18) | 0.53  (0.16-1.79) |
| **Income group** |  |  |  |  |  |  |  |  |  |  |
| ABC1 | 1 (Ref) | 1 (Ref) | 1 (Ref) | 1 (Ref) | 1 (Ref) | 1 (Ref) | 1 (Ref) | 1 (Ref) | 1 (Ref) | 1 (Ref) |
| C2DE | 0.82  (0.42-1.59) | 1.11  (0.44-2.83) | 0.77  (0.46-1.30) | 1.10  (0.50-2.43) | 0.65  (0.40-1.07) | 0.82  (0.42-1.59) | **0.66**  **(0.46-0.95)** | 0.86  (0.50-1.50) | 1.11  (0.51-2.42) | 1.63  (0.51-5.18) |
| **BMI (5 kg/m^2^)** | 1.33  (0.91-1.93) | **1.64**  **(1.04-2.61)** | 1.12  (0.84-1.48) | 1.16  (0.81-1.66) | 1.37  (1.04-1.80) | 1.33  (0.95-1.88) | **1.34**  **(1.08-1.67)** | 1.25  (0.96-1.63) | 1.15  (0.77-1.71) | 1.17  (0.70-1.96) |
| **Smoking status** |  |  |  |  |  |  |  |  |  |  |
| Non-smoker | 1 (Ref) | 1 (Ref) | 1 (Ref) | 1 (Ref) | 1 (Ref) | 1 (Ref) | 1 (Ref) | 1 (Ref) | 1 (Ref) | 1 (Ref) |
| Ex-smoker | 0.57  (0.32-1.04) | 0.93  (0.43-2.03) | 0.72  (0.46-1.13) | 0.96  (0.52-1.78) | 1.07  (0.68-1.69) | 1.78  (0.95-3.35) | 0.95  (0.68-1.33) | 1.22  (0.76-1.95) | 0.73  (0.39-1.38) | 1.77  (0.68-4.57) |
| Current smoker | 0.52  (0.26-1.06) | 1.67  (0.56-4.97) | 0.93  (0.51-1.73) | 2.55  (0.85-7.60) | 0.73  (0.43-1.24) | 1.32  (0.61-2.86) | 0.97  (0.63-1.50) | 1.15  (0.61-2.14) | 0.73  (0.33-1.62) | 1.38  (0.39-4.84) |
| **Beliefs about cancer outcomes** | **1.44**  **(1.05-1.97)** | **1.49**  **(1.02-2.17)** | **1.35**  **(1.05-1.75)** | 1.37  (0.99-1.90) | 1.26  (0.99-1.59) | 1.35  (0.99-1.83) | 1.19  (0.99-1.42) | 1.14  (0.89-1.46) | 1.26  (0.95-1.67) | 1.20  (0.77-1.88) |
| **Beliefs about cancer treatment** | 1.20  (0.90-1.59) | 1.07  (0.71-1.60) | 1.13  (0.90-1.42) | 0.98  (0.71-1.36) | 1.20  (0.98-1.46) | 0.95  (0.70-1.29) | 1.10  (0.94-1.28) | 0.91  (0.72-1.16) | 1.37  (1.01-1.85) | 1.23  (0.78-1.96) |
| **Cancer worry** | 1.05  (0.93-1.19) | 1.13  (0.95-1.34) | **1.11**  **(1.00-1.24)** | **1.24**  **(1.05-1.47)** | **1.13**  **(1.02-1.26)** | **1.18**  **(1.02-1.37)** | **1.12**  **(1.04-1.20)** | **1.17**  **(1.05-1.32)** | **1.22**  **(1.01-1.45)** | 1.27  (0.98-1.64) |
| **Perceived cancer risk** | 0.99  (0.98-1.01) | **0.98**  **(0.96-0.99)** | 1.01  (0.99-1.02) | 0.99  (0.99-1.90) | 1.01  (0.99-1.02) | 1.00  (0.98-1.01) | **1.01**  **(1.00-1.01)** | 1.00  (0.98-1.01) | 1.02  (0.99-1.03) | 1.00  (0.98-1.02) |
| **Burden/inconvenience associated with the test** | **0.36**  **(0.23-0.55)** | **0.40**  **(0.23-0.70)** | **0.28**  **(0.21-0.38)** | **0.38**  **(0.25-0.58)** | **0.39**  **(0.31-0.50)** | **0.46**  **(0.33-0.64)** | **0.28**  **(0.23-0.34)** | **0.38**  **(0.29-0.51)** | **0.55**  **(0.41-0.76)** | 0.64  (0.37-1.10) |
| **Worry associated with the test** | **0.66**  **(0.49-0.89)** | 1.00  (0.58-1.70) | **0.48**  **(0.39-0.59)** | **0.60**  **(0.42-0.85)** | **0.60**  **(0.48-0.75)** | 0.74  (0.53-1.04) | **0.42**  **(0.35-0.50)** | **0.62**  **(0.48-0.80)** | **0.63**  **(0.49-0.82)** | 0.91  (0.54-1.51) |
