# Supplementary material for: Public attitudes towards screening for kidney cancer: an online survey
Source: BMC Urol. 2020 Oct 28;20:170. doi: 10.1186/s12894-020-00724-0 (PMC7592501; doi:10.1186/s12894-020-00724-0)
Supplement: Supplementary file 4 — Additional file 4. Supplementary Figures. [file 12894_2020_724_MOESM4_ESM.pptx]

## Slide 1
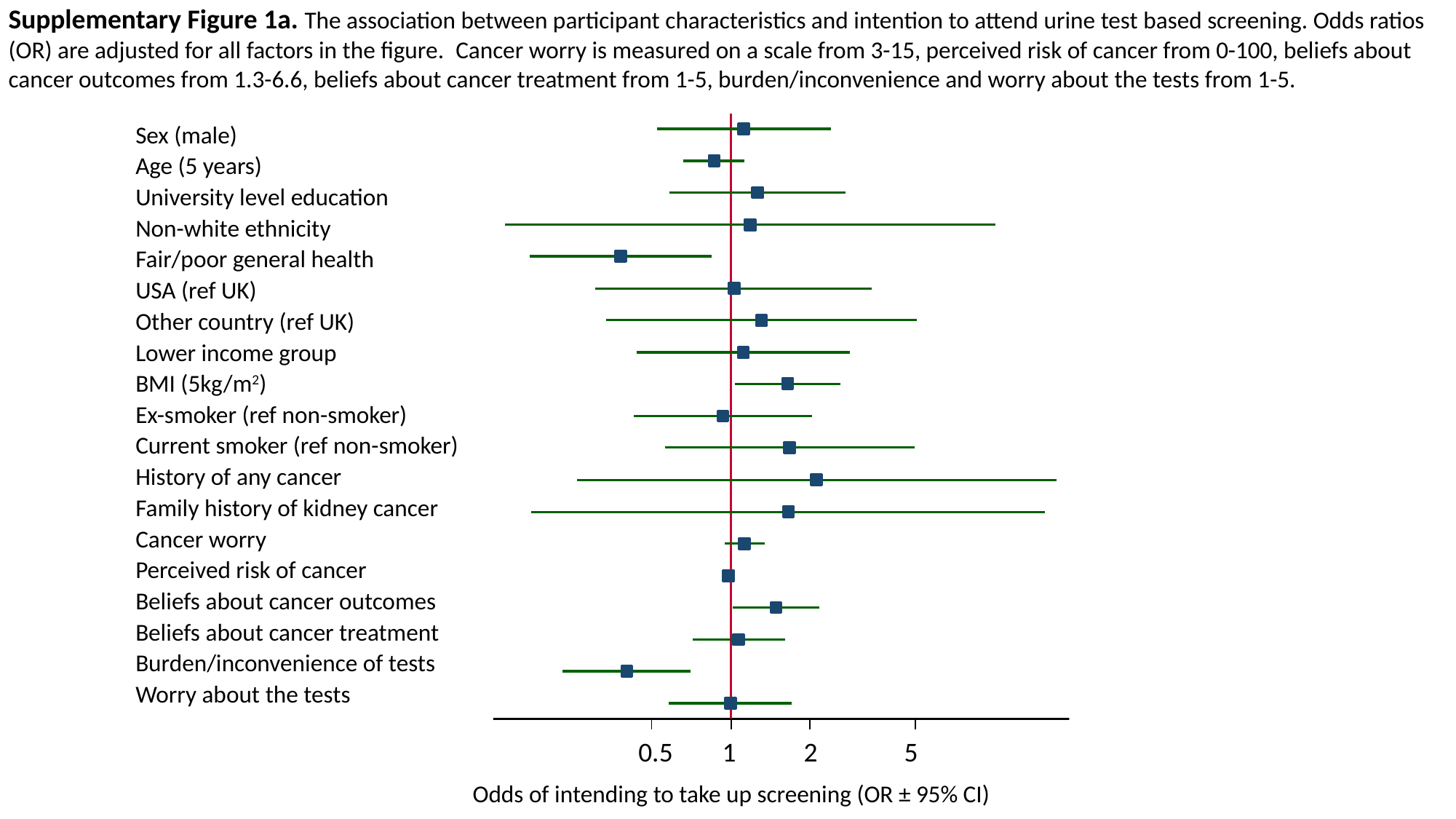

Supplementary Figure 1a. The association between participant characteristics and intention to attend urine test based screening. Odds ratios (OR) are adjusted for all factors in the figure.  Cancer worry is measured on a scale from 3-15, perceived risk of cancer from 0-100, beliefs about cancer outcomes from 1.3-6.6, beliefs about cancer treatment from 1-5, burden/inconvenience and worry about the tests from 1-5.
Sex (male)
Age (5 years)
University level education
Non-white ethnicity
Fair/poor general health
USA (ref UK)
Other country (ref UK)
Lower income group
BMI (5kg/m2)
Ex-smoker (ref non-smoker)
Current smoker (ref non-smoker)
History of any cancer
Family history of kidney cancer
Cancer worry
Perceived risk of cancer
Beliefs about cancer outcomes
Beliefs about cancer treatment
Burden/inconvenience of tests
Worry about the tests
0.5 1 2 5
Odds of intending to take up screening (OR ± 95% CI)

## Slide 2
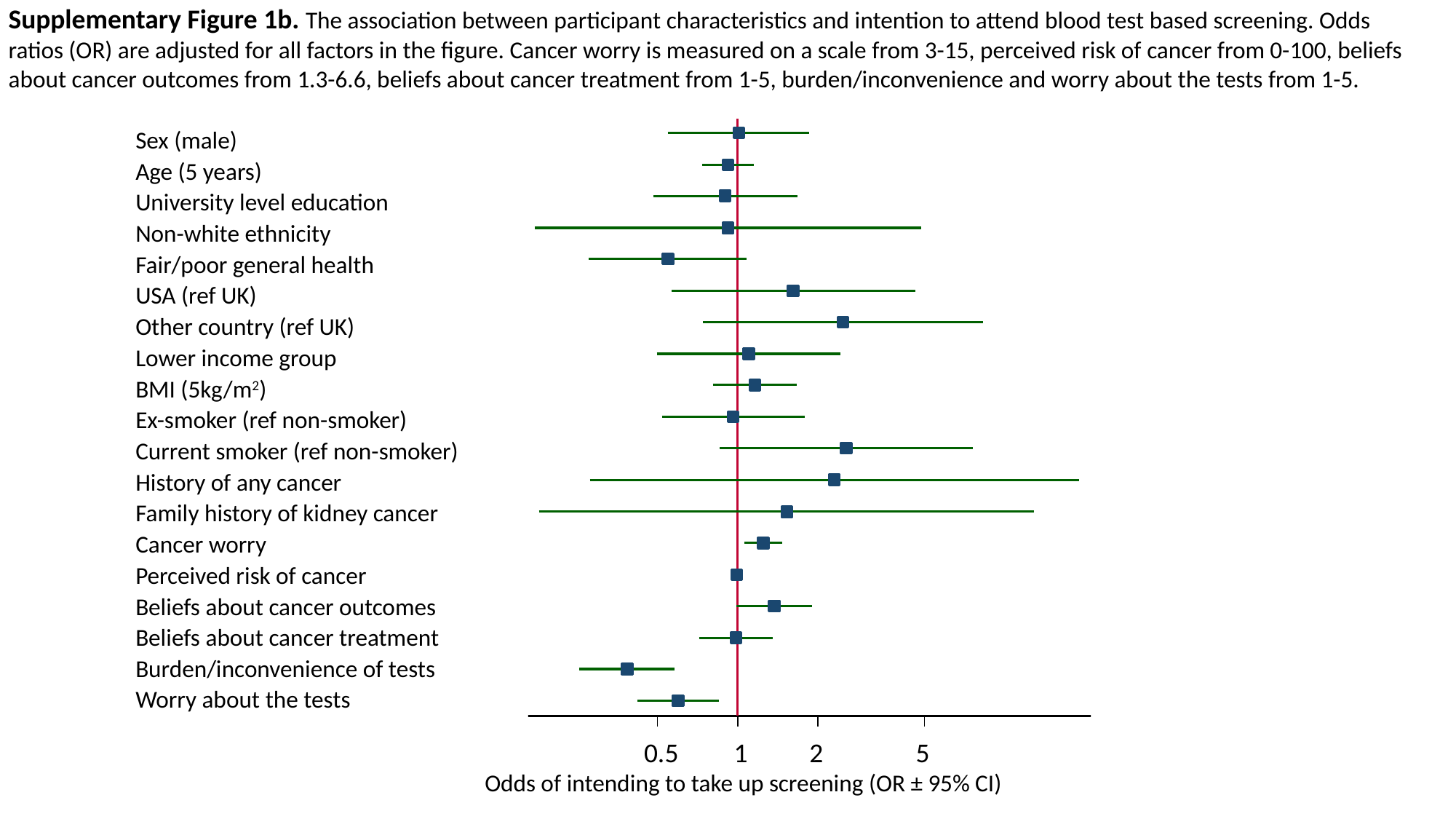

Supplementary Figure 1b. The association between participant characteristics and intention to attend blood test based screening. Odds ratios (OR) are adjusted for all factors in the figure. Cancer worry is measured on a scale from 3-15, perceived risk of cancer from 0-100, beliefs about cancer outcomes from 1.3-6.6, beliefs about cancer treatment from 1-5, burden/inconvenience and worry about the tests from 1-5.
Sex (male)
Age (5 years)
University level education
Non-white ethnicity
Fair/poor general health
USA (ref UK)
Other country (ref UK)
Lower income group
BMI (5kg/m2)
Ex-smoker (ref non-smoker)
Current smoker (ref non-smoker)
History of any cancer
Family history of kidney cancer
Cancer worry
Perceived risk of cancer
Beliefs about cancer outcomes
Beliefs about cancer treatment
Burden/inconvenience of tests
Worry about the tests
0.5 1 2 5
Odds of intending to take up screening (OR ± 95% CI)

## Slide 3
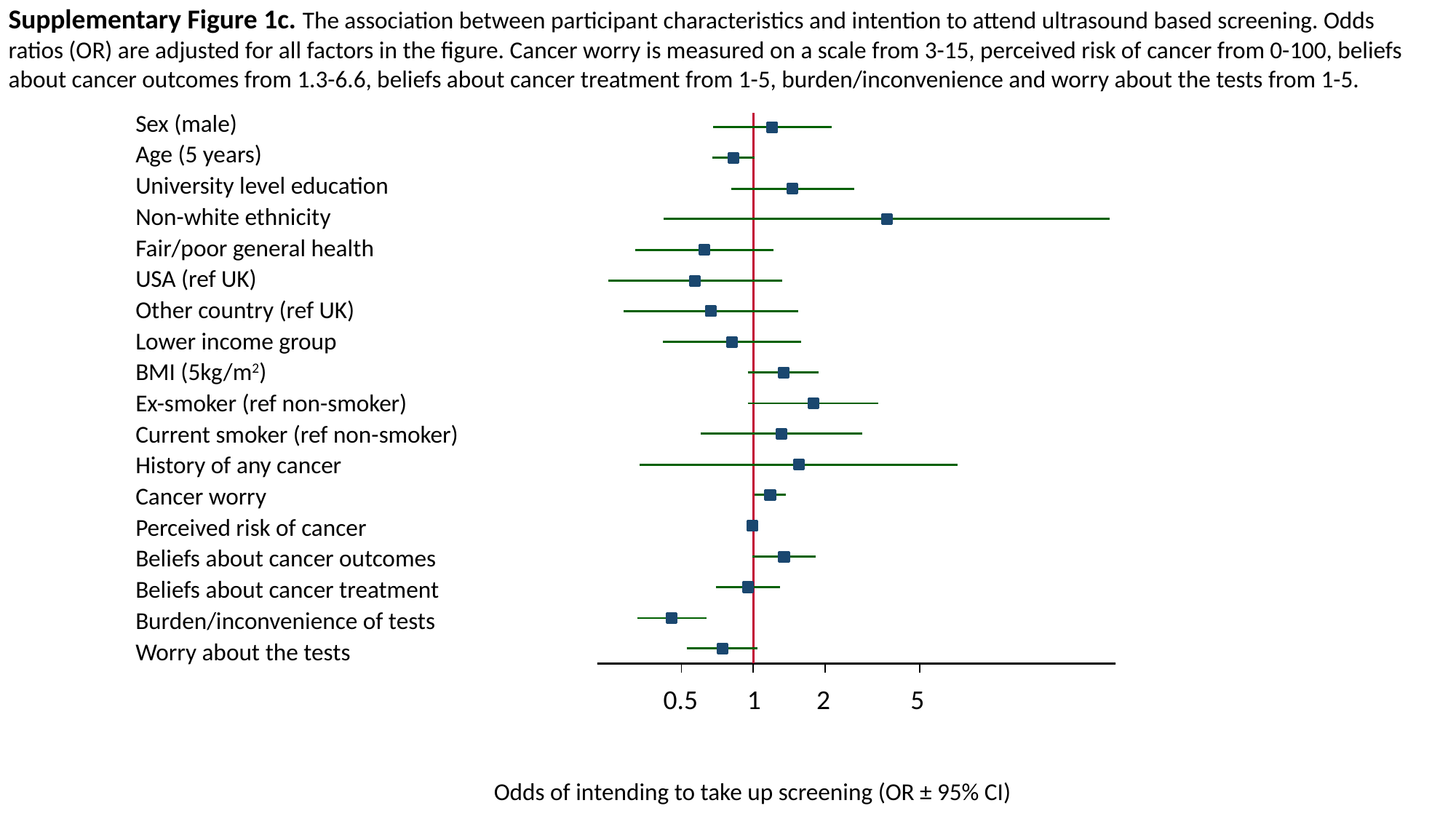

Supplementary Figure 1c. The association between participant characteristics and intention to attend ultrasound based screening. Odds ratios (OR) are adjusted for all factors in the figure. Cancer worry is measured on a scale from 3-15, perceived risk of cancer from 0-100, beliefs about cancer outcomes from 1.3-6.6, beliefs about cancer treatment from 1-5, burden/inconvenience and worry about the tests from 1-5.
Sex (male)
Age (5 years)
University level education
Non-white ethnicity
Fair/poor general health
USA (ref UK)
Other country (ref UK)
Lower income group
BMI (5kg/m2)
Ex-smoker (ref non-smoker)
Current smoker (ref non-smoker)
History of any cancer
Cancer worry
Perceived risk of cancer
Beliefs about cancer outcomes
Beliefs about cancer treatment
Burden/inconvenience of tests
Worry about the tests
0.5 1 2 5
Odds of intending to take up screening (OR ± 95% CI)

## Slide 4
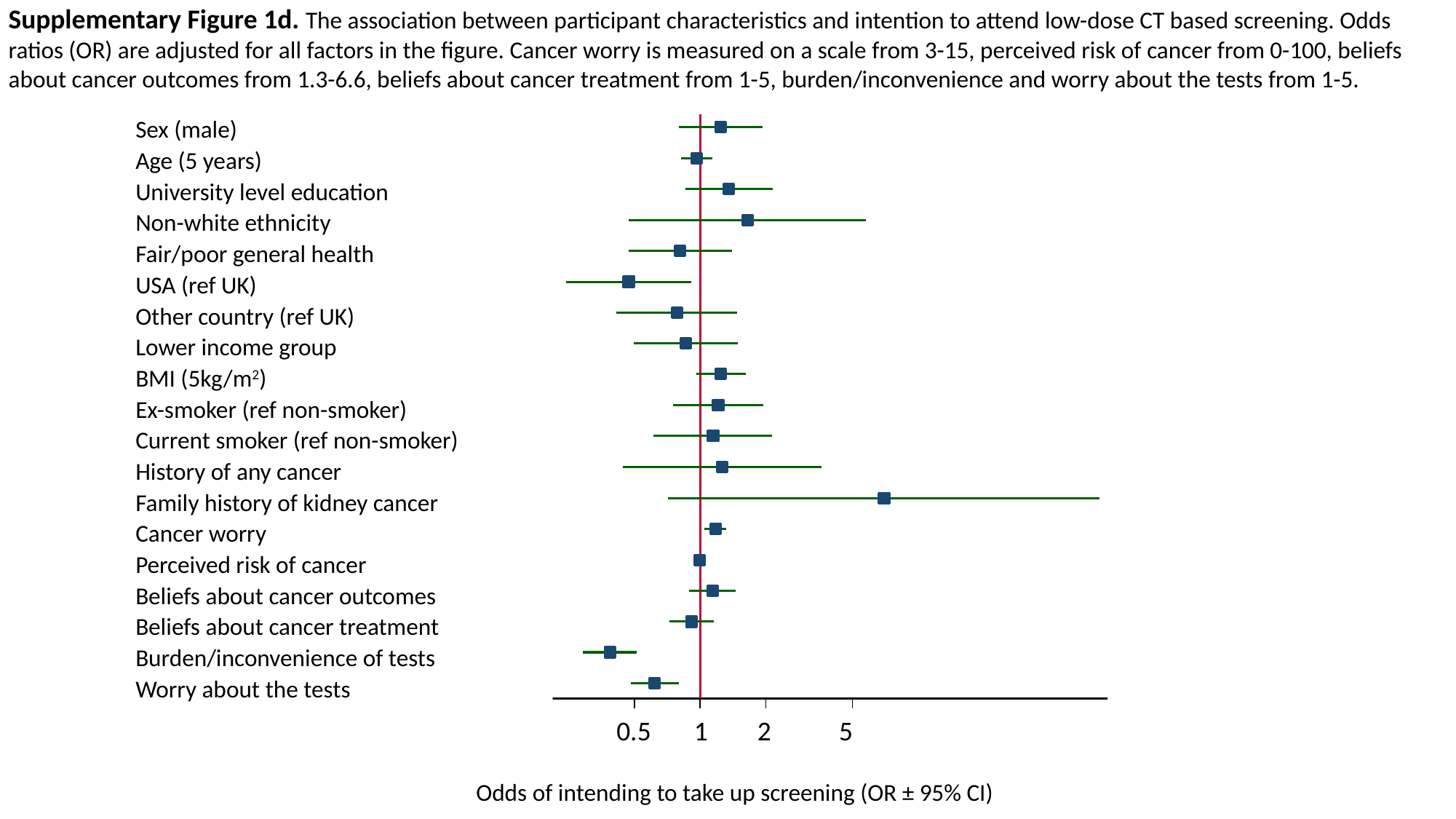

Supplementary Figure 1d. The association between participant characteristics and intention to attend low-dose CT based screening. Odds ratios (OR) are adjusted for all factors in the figure. Cancer worry is measured on a scale from 3-15, perceived risk of cancer from 0-100, beliefs about cancer outcomes from 1.3-6.6, beliefs about cancer treatment from 1-5, burden/inconvenience and worry about the tests from 1-5.
Sex (male)
Age (5 years)
University level education
Non-white ethnicity
Fair/poor general health
USA (ref UK)
Other country (ref UK)
Lower income group
BMI (5kg/m2)
Ex-smoker (ref non-smoker)
Current smoker (ref non-smoker)
History of any cancer
Family history of kidney cancer
Cancer worry
Perceived risk of cancer
Beliefs about cancer outcomes
Beliefs about cancer treatment
Burden/inconvenience of tests
Worry about the tests
0.5 1 2 5
Odds of intending to take up screening (OR ± 95% CI)

## Slide 5
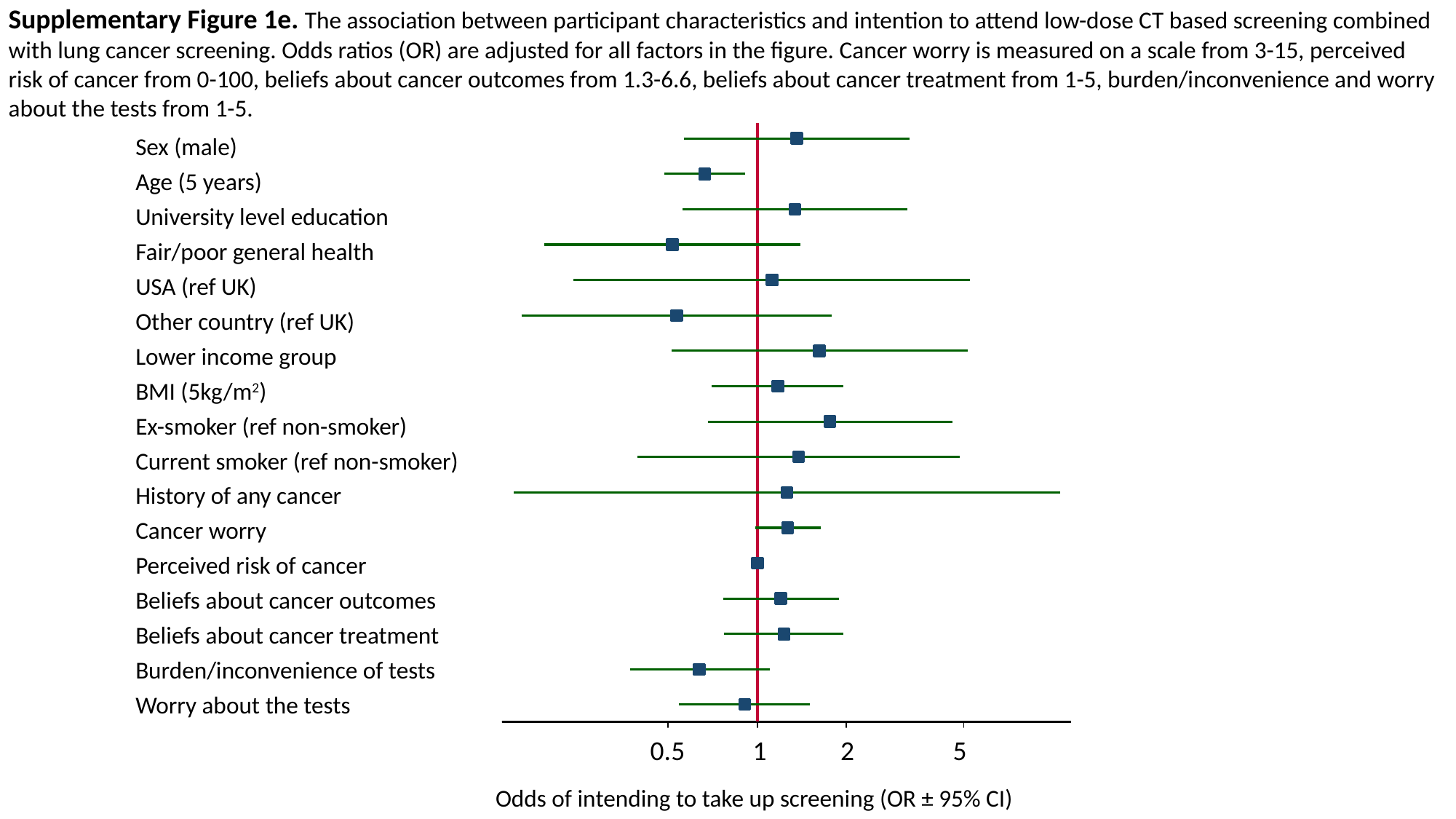

Supplementary Figure 1e. The association between participant characteristics and intention to attend low-dose CT based screening combined with lung cancer screening. Odds ratios (OR) are adjusted for all factors in the figure. Cancer worry is measured on a scale from 3-15, perceived risk of cancer from 0-100, beliefs about cancer outcomes from 1.3-6.6, beliefs about cancer treatment from 1-5, burden/inconvenience and worry about the tests from 1-5.
Sex (male)
Age (5 years)
University level education
Fair/poor general health
USA (ref UK)
Other country (ref UK)
Lower income group
BMI (5kg/m2)
Ex-smoker (ref non-smoker)
Current smoker (ref non-smoker)
History of any cancer
Cancer worry
Perceived risk of cancer
Beliefs about cancer outcomes
Beliefs about cancer treatment
Burden/inconvenience of tests
Worry about the tests
0.5 1 2 5
Odds of intending to take up screening (OR ± 95% CI)
